# Physiotherapy as a Rare Cause of Twiddler’s Syndrome in a Patient With an Implanted Cardioverter Defibrillator

**DOI:** 10.4021/cr260w

**Published:** 2013-05-09

**Authors:** Christiana Schernthaner, Franz Danmayr, Richard Krausler, Bernhard Strohmer

**Affiliations:** a Department of Cardiology, Salzburger Landeskliniken, Paracelsus Private Medical University Salzburg, Austria; b Department of Cardiac Surgery, Salzburger Landeskliniken, Paracelsus Private Medical University Salzburg, Austria

**Keywords:** Twiddler Syndrom, Implantable cardioverter defibrillator, Lead dysfunction, Physiotherapy

## Abstract

A 65-year-old male patient with a history of ischemic cardiomyopathy developed ventricular tachycardia resulting in presyncope. An ICD was indicated for secondary prophylaxis of ventricular tachyarrhythmias. A dual chamber ICD was implanted from the right side because insertion of the device from the left side was unfeasible after surgery of a left subscapularis tendon lesion. ICD implantation and testing of defibrillation threshold were uneventful. During early follow-up a progressive increase of the stimulation threshold was detected. On chest X-ray coiling of both atrial and ventricular leads was noted and caused inadvertently by active shoulder-arm physiotherapy. Complete revision of the ICD system was necessary for restoration of the pacemaker function of the ICD. This unique case highlights important steps for early recognition and prevention of Twiddler’s syndrome that may occur due to physiotherapy treatment even without abnormal manipulations by the patient.

## Introduction

The Twiddler’s syndrome is a rarely reported complication in recipients of an implantable cardioverter defibrillator (ICD). Only a few case reports exist about ICD dysfunction, inappropriate shocks and lead retraction due to the coiling of the leads in the subfascial and subpectoral position [1-7]. The Twiddler’s syndrome was first described in patients implanted with pacemakers [8]. A number of risk factors have been related to the occurrence of the Twiddler’s syndrome, such as oversized device pockets, atrophy of the pectoralis muscle with subpectoral implantation, and low weight impulse generators [2, 3, 5-7]. The following report summarizes the subtle findings of this unusual complication in a patient with a right sided dual chamber ICD system undergoing physiotherapy.

## Case Report

A 65-year-old male patient was admitted to the neurological care unit because of presyncope suspicious for a cerebrovascular ischemic accident. During the monitoring phase the patient developed a rapid sustained ventricular tachycardia mimicking again neurological symptoms. Structural heart disease was present in form of ischemic cardiomyopathy with an ejection fraction of 0.40 and a scar in the inferior region. There was a history of coronary artery bypass revascularisation 14 years ago, but no signs of acute coronary syndrome. Recent coronary angiography demonstrated severe three vessel disease with a patent arterial graft to the left anterior descending artery, an open vein graft to the circumflex artery, an occluded right coronary artery with an occluded single vein graft, but no need for further revascularization. A week before hospital admission the patient underwent surgery for rupture of the left sided tendon of the subscapularis muscle and a surgical revision due to wound hematoma.

**Figure 1:**
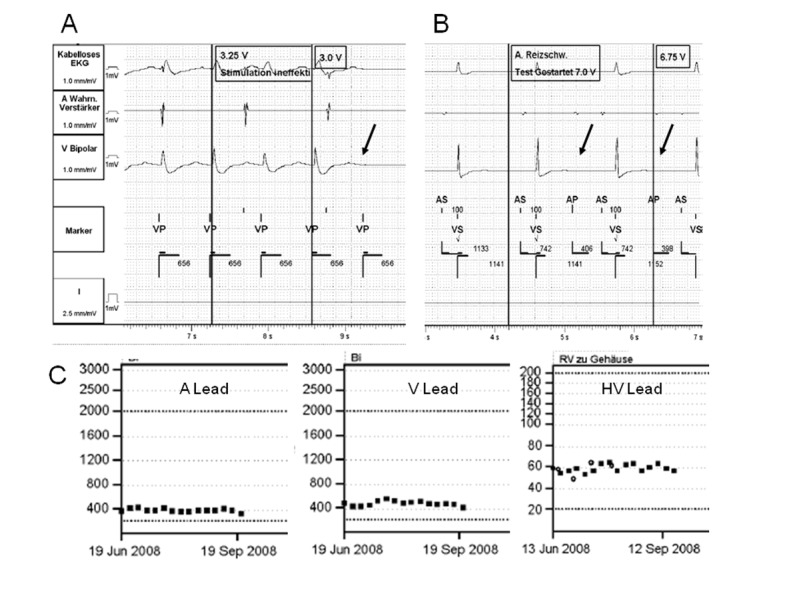
(A) The printout shows loss of capture at 3.0 V @ 0.5 ms during ventricular threshold testing suggestive of subacute increase of pacing threshold in ischemic cardiomyopathy. (B) Strip shows no capture of atrial pacing at maximum output of 7.0 V together with clearly reduced amplitude of the intrinsic atrial signal due to farfield sensing of the retracted electrode. (C) The impedance values of the atrial, ventricular and shock lead remained within the normal range despite the threshold problems.

For secondary prevention of ventricular tachyarrhythmias a dual-chamber ICD (St. Jude Medical Current DR RF, 2207-36; atrial lead SJM 1888TC/52; ventricular lead SJM Durata 7170) was implanted from the right side without any problems. The operation was performed in the usual way. The greater pectoral muscle was divided between its subclavian and thoracic part in order to form a pocket beyond the pectoralis muscle. The ventricular and atrial leads were introduced after direct puncture of the subclavian vein and adequate sensing, pacing and impedance values were confirmed (R wave amplitude 5.7 mV, threshold 0.9 V at 0.5 ms, impedance 872 Ohm; P wave amplitude 3.9 mV, threshold 0.8 V at 0.5 ms, impedance 764 Ohm). The leads were secured with non-absorbable double sutures around the sleeves. The wound was closed after placement of the ICD generator into the subpectoral pocket. Defibrillation testing was performed successfully with a single 17 Joule shock. At prehospital discharge control the patient claimed about intermittent stimulation of the diaphragm. Testing of ventricular threshold revealed an increase to 3.0 V at 0.5 ms while impedance and sensing values remained normal (Fig. 1A). The pacing mode was changed to AAI stimulation to prevent the unpleasant diaphragmatic stimulation. Within the first three months after implantation the patient received a total of 8 adequate antitachycardia pacing therapies and 3 defibrillator discharges at maximum output for treatment of ventricular fibrillation. Despite the life saving effect the patient was worried by the painful therapies of the ICD.

**Figure 2:**
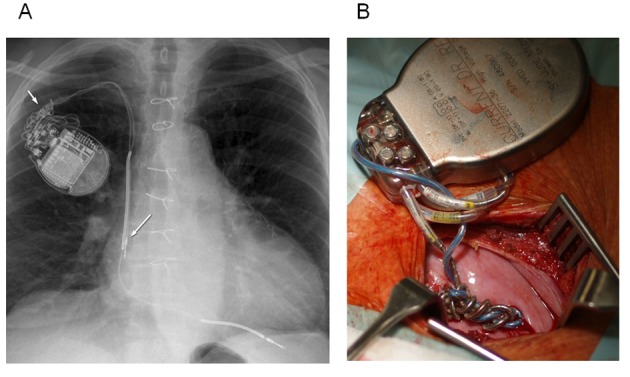
(A) The chest X-ray was indicative of Twiddler’s syndrome demonstrating retraction of the atrial lead and excessive coiling near the ICD header (see arrows). (B) Image taken during surgical revision of the ICD system showing coiled leads in situ still connected to the ICD.

At 3-months follow-up the patient demonstrated a further increase of the ventricular stimulation threshold up to 5.0 V and a complete loss of capture of the atrial lead with diminished atrial amplitude during sensing (Fig. 1B). Interestingly, no changes were noted with respect to the impedance values of the atrial, ventricular and high-voltage shock lead (Fig. 1C). The local aspect of the ICD pocket was unremarkable. A chest X-ray revealed retraction of the atrial lead up to the superior caval vein and extreme coiling of the leads near the header (Fig. 2A). The patient underwent a complete revision of the ICD system. Upon opening the pocket the surgeon found an atrophy of the pectoralis muscle and heavily tangled leads without isolation defect (Fig. 2B). After uncoiling the leads the ventricular stimulation threshold was within the normal range again. However, both atrial and ventricular leads were extracted and replaced by new ones for safety reasons. Before closing the pocket the generator was secured with a suture to the pectoralis muscle.

## Discussion

The present case demonstrates the nonspecific findings of Twiddler’s syndrome in the early phase after a right-sided ICD implantation. The initial observations mimicked a subacute increase of the stimulation threshold of the atrial and ventricular leads that was compensated by reprogramming of the output. It is of interest, that monitoring of impedance and intrinsic signals was not helpful to detect the chronic lead retraction. Inspection of the ICD pocket did not give any clue about presence of a Twiddler’s syndrome. The chest X-ray turned out to be a critical diagnostic measure for detection of this specific complication during early follow-up.

The patient has a combination of several risk factors for development of the Twiddler’s syndrome, such as hospital immobilisation and atrophy of the pectoralis muscle [3]. According to literature subpectoral implantation has been recognized to facilitate the mobility of the implanted generator [1, 5, 9]. The patient was bothered by the painful perception of antitachycardia pacing related to phrenic nerve stimulation. From time to time the patient checked the position of his device but denied to have manipulated extensively. It is noteworthy, that all daily activities had to be managed with the right arm due to surgery and immobilization of the left shoulder. It is conceivable that the increased muscular activity of the right shoulder and arm might have led to repetitive motions within the pocket favouring the coiling of the leads [2, 3]. In a recent report hard physical training during neurological rehabilitation has been associated with the twiddling of the proximal transvenous defibrillation lead [10].

There is evidence that Twiddler’s syndrome may occur in patients undergoing active physiotherapy of the shoulder-arm region, even without external manipulation by the patient himself. The present case represents such a form of inadvertent, exercise-induced Twiddler’s syndrome and highlights the importance of educating physiotherapists as well as patients on the type of exercise they can perform safely. In addition, early recognition of pacemaker dysfunction and appropriate implantation technique may help to prevent the occurrence of this syndrome requiring almost always surgical revision [11].
